# A mission to understand space anaemia

**DOI:** 10.1038/s43856-022-00078-8

**Published:** 2022-02-15

**Authors:** Ben Abbott

**Affiliations:** Communications Medicine, https://www.nature.com/commsmed

## Abstract

Space flight takes its toll on the human body. A recent study in *Nature Medicine* sought to characterise the physiological mechanisms through which anaemia arises in astronauts on long-duration space flight.

**Figure:**
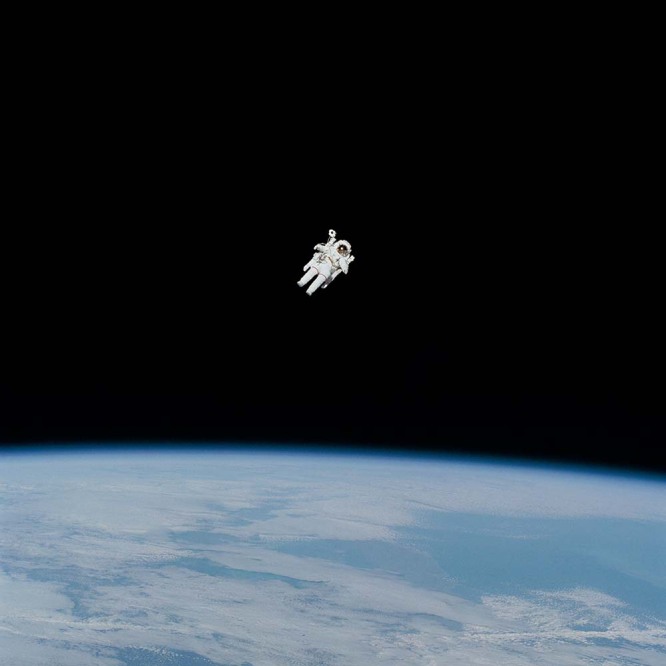
NASA on Unsplash

With astronauts engaged in longer missions to space and the ongoing commercialisation of space travel making space accessible to larger numbers of people, there is a growing need to understand the impact of space flight on human health.

The phenomenon of space anaemia—whereby red blood cell (RBC) mass is reduced during space travel—has long been observed in astronauts, although has largely been thought to be a transient adaptation to low-gravity conditions. The mechanisms underpinning space anaemia, as well as whether space flight leads to longer-term effects on RBCs, have not yet been established.

A recent study by Trudel and colleagues examined the physiological mechanisms of anaemia in astronauts during and after long-duration space flight^1^. The study recruited 14 astronauts and collected serial blood and exhaled air samples over their 6-month missions aboard the International Space Station, and for 1 year after their return. These samples were used to measure markers of the destruction of RBCs, known as haemolysis, including carbon monoxide (CO) elimination and free haemoglobin levels in the blood.

The authors report increased CO elimination 5 days into the mission, compared to pre-mission levels, and values remained raised over the several months spent in space. Haemoglobin levels were also raised, and there were signs of a compensatory increase in RBC production. Importantly, haemolysis and haemoglobin levels remained raised 1 year after returning from space, indicating a longer-term effect of space flight on RBCs.

In summary, these findings suggest that anaemia arising in those who travel to space is a result of the destruction of RBCs, and might have important implications for monitoring the health of space travellers during and after space flight, as well as determining who is suitable for space travel.
